# High Photon‐to‐Current Conversion in Solar Cells Based on Light‐Absorbing Silver Bismuth Iodide

**DOI:** 10.1002/cssc.201700634

**Published:** 2017-06-01

**Authors:** Huimin Zhu, Mingao Pan, Malin B. Johansson, Erik M. J. Johansson

**Affiliations:** ^1^ Department of Chemistry- Ångström, Div. Physical ChemistryUppsala UniversityBox 523SE-751 20UppsalaSweden

**Keywords:** perovskite solar cells, power conversion efficiency, silver bismuth iodide, space group, x-ray diffraction

## Abstract

Here, a lead‐free silver bismuth iodide (AgI/BiI_3_) with a crystal structure with space group *R* $\bar{3}$ *m* is investigated for use in solar cells. Devices based on the silver bismuth iodide deposited from solution on top of TiO_2_ and the conducting polymer poly(3‐hexylthiophene‐2,5‐diyl) (P3HT) as a hole‐transport layer are prepared and the photovoltaic performance is very promising with a power conversion efficiency over 2 %, which is higher than the performance of previously reported bismuth‐halide materials for solar cells. Photocurrent generation is observed between 350 and 700 nm, and the maximum external quantum efficiency is around 45 %. The results are compared to solar cells based on the previously reported material AgBi_2_I_7_, and we observe a clearly higher performance for the devices with the new silver and bismuth iodides composition and different crystal structure. The X‐ray diffraction spectrum of the most efficient silver bismuth iodide material shows a hexagonal crystal structure with space group *R* $\bar{3}$ *m*, and from the light absorption spectrum we obtain an indirect band gap energy of 1.62 eV and a direct band gap energy of 1.85 eV. This report shows the possibility for finding new structures of metal‐halides efficient in solar cells and points out new directions for further exploration of lead‐free metal‐halide solar cells.

Lead‐halide perovskite solar cells (PSC) have shown a tremendous development the recent years. In 2009, CH_3_NH_3_PbI_3_ was used as a light absorber in a liquid dye‐sensitized solar cell (DSC), which resulted in a power conversion efficiency (PCE) of 3.8 %.^1^ The perovskite material was later used in a solid‐state solar cell and a PCE around 10 % was obtained, which was a breakthrough in the solar cell research field.^2^, ^3^ After a rapid development, an efficiency of over 20 % has been achieved.^4^ These results are therefore very promising for future application of perovskite solar cells in electricity production. However, lead is toxic, which might be an issue for a very large‐scale application of the lead‐halide perovskite.^5^ Therefore, other elements have been explored to replace lead, and perovskites based on tin (Sn) show promising efficiencies.^6^, ^7^ However, Sn perovskites are rather unstable and their toxicity is not clear at the moment.^5^

Bismuth is another low‐toxic alternative for replacement of lead and it was shown that it is possible to make functional solar cells with bismuth‐halide perovskites with a similar device structure as the lead perovskite solar cells.^8^ It was also later shown that bismuth‐halide perovskite‐based solar cells can be prepared with different device structures, and using different stoichiometry of the ions.^9^, ^10^, ^11^, ^12^, ^13^, ^14^, ^15^, ^16^, ^17^, ^18^ Calculations of some of these bismuth‐halide‐based perovskites, show however that the electronic structure might not be optimal for charge transport in three dimensions in some of these materials.^19^, ^20^ Also, bismuth‐halide double perovskites with, for example, silver ions (Ag^+^) included in the structure have been investigated, which also show promising optical properties.^21^, ^22^, ^23^, ^24^, ^25^ However, theoretical calculations of the double perovskite bismuth halides show that the double perovskites also may have rather low charge mobilities.^26^ Therefore, other structures of bismuth halides may be more advantageous in solar cells and recently it was reported that the silver bismuth‐halide material AgBi_2_I_7_ functions as a photovoltaic material, and solar cells with over 1 % PCE could be obtained.^27^ Moreover, BiI_3_ shows promising efficiency in solar cells^28^ and other bismuth‐halide materials show interesting properties that may be useful in solar cells.^29^ A screening of different Ag_1‐3*x*_Bi_1+*x*_I4 (0<*x*<0.19) compounds has previously been performed, indicating that there exist several stable compositions and the structure is not trivial owing to the vacancies in the system.^30^

Here, solutions with a mixture of silver iodide (AgI) and bismuth iodide (BiI_3_) with molar ratio of AgI/BiI_3_=2:1 and AgI/BiI_3_=1:2 were used to synthesize materials with different crystal structures. These materials were used in a solar cell between TiO_2_, which acts as electron‐conducting layer, and poly(3‐hexylthiophene‐2,5‐diyl) (P3HT), which is used as hole‐transport layer. The PCE of the best devices was above 2 % under 1000 W m^−2^ (1 sun), AM 1.5 G simulated solar illumination. UV/Vis spectroscopy, X‐ray diffraction (XRD), and scanning electron microscopy (SEM) were used to investigate the properties of the material, and incident photon‐to‐current conversion efficiency (IPCE) and transient photovoltage decay measurements were used to try to understand the light‐induced processes in the solar cell. The results were also compared to results from devices with the previously reported material AgBi_2_I_7_.

The crystal structures of the silver bismuth iodides made from solutions with different ratios of AgI and BiI_3_ were analyzed by XRD and the results are shown in Figure 1. Figure 1 d shows the diffraction pattern of the AgI/BiI_3_ samples made from solutions with molar ratio 1:2 with a layer of the hole‐transport material P3HT on the silver bismuth iodide material, Figure 1 e shows AgI/BiI_3_ samples made from solutions with molar ratio 1:2 without P3HT, Figure 1 f shows AgI/BiI_3_ samples made from solutions with molar ratio 2:1 with a layer of P3HT, and Figure 1 g shows AgI/BiI_3_ samples made from solutions with molar ratio 2:1 without P3HT. The hole‐transport material P3HT protects the sample from degradation, which is clearly seen in the intensity difference of the diffraction peaks when comparing the samples with and without P3HT in Figure 1 d, e as well as in Figure 1 f, g. The sample with the 2:1 molar ratio solution in Figure 1 f matches very well a hexagonal (trigonal) structure with the space group *R* $\bar{3}$ *m* and cell parameters of *a*=4.350 Å and *c*=20.82 Å.^29^ The diffraction planes for the strongest diffraction peaks are marked in the Figure.^29^, ^31^ The highest intensity comes from the (104) and (003) planes corresponding to a spacing of *d=*3.06 Å and *d=*6.97 Å, respectively. The sample from a solution with molar ratio 1:2 forms a cubic structure with space group *Fd* $\bar{3}$ *m* with an approximate cell parameter of *a=*12.223 Å. It is uncertain if the 1:2 molar ratio solution forms AgBi_2_I_7_ or a substoichiometric Ag‐deficient AgBiI_4_ structure. Mashadieva et al.^29^ reported on AgBi_2_I_7_, which was calculated based on electro‐motive force (EMF) measurements. Recently, Xiao et al.^32^ investigated the AgBi_2_I_7_ material and suggested that it cannot be formed in the ThZr_2_H_7_‐type structure, because the Bi−I bond length is too short and it may result in an unreasonably large mass density. Instead, it was suggested that the cubic AgBiI_4_ is formed with the lattice constant *a=*12.2223 Å. Moreover, the theoretically calculated AgBi_2_I_7_ has a much higher intensity of the (440) plane (*d=*2.16 Å), whereas the theoretically calculated AgBiI_4_ has a much higher intensity of the (400) plane, corresponding to *d=*3.05 Å. Herein, the (400) plane has the highest intensity, however, we cannot state which cubic structure is formed as the AgBiI_4_ and AgBi_2_I_7_ structures are very similar. A clear difference between the samples with space group *Fd* $\bar{3}$ *m* and *R* $\bar{3}$ *m* is observed at 2*θ=*42°, where *Fd* $\bar{3}$ *m* has only one diffraction peak from the (440) plane, and the *R* $\bar{3}$ *m* structure has two diffraction peaks from the (110) and (108) planes (Figure S1 in the Supporting Information). We can therefore clearly distinguish the samples made from molar ratio solutions AgI/BiI_3_=1:2 and AgI/BiI_3_=2:1 by the space group difference. The previously reported structures for AgBiI_4_ and AgBi_2_I_7_ have the same *Fd* $\bar{3}$ *m* space group, which we also observe here for the material with molar ratio solution AgI/BiI_3_=1:2, whereas we observe a structure with space group *R* $\bar{3}$ *m* for the material from molar ratio solution AgI/BiI_3_=2:1. The space group *R* $\bar{3}$ *m* is in agreement with the crystal structure of Ag_2_BiI_5_, which we expect from the molar ratio in the solution. Here, we therefore hereafter use the space group to name the samples made from different molar ratio solutions. The diffraction peaks addressed with a star are in agreement with previously observed diffraction peaks in hexagonal Bi compositions.^15^ The samples are stable during the XRD measurements and during storage in the N_2_ environment and they showed the same XRD patterns after 7 days (see Figure S3).

**Figure 1:**
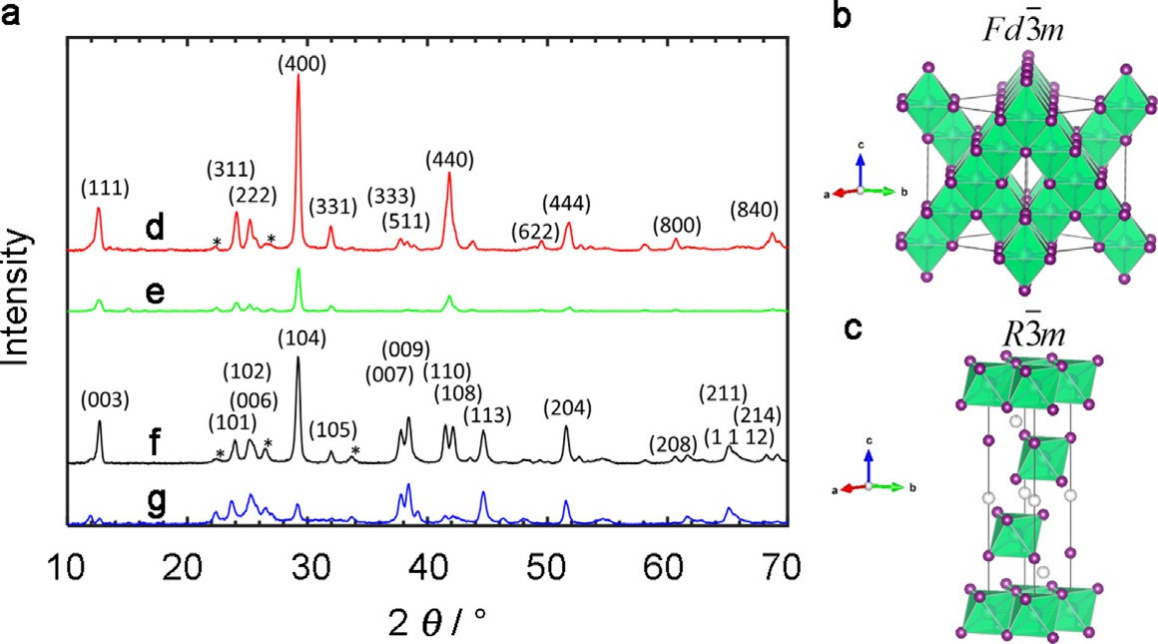
a) XRD patterns of the different samples, b) schematic of a cubic crystal structure with space group *Fd* $\bar{3}$ m, c) schematic of a hexagonal (trigonal) structure with space group *R* $\bar{3}$ m, d) XRD pattern of the sample with molar ratio AgI/BiI_3_=1:2 with space group *Fd* $\bar{3}$ *m* with a layer of P3HT on top, e) XRD pattern of the sample with molar ratio AgI/BiI_3_=1:2 with space group *Fd* $\bar{3}$ *m* without P3HT, f) XRD pattern of the sample with molar ratio AgI/BiI_3_=2:1 with space group *R* $\bar{3}$ *m* with a layer of P3HT on top, g) XRD pattern of the sample with molar ratio AgI/BiI_3_=2:1 with space group *R* $\bar{3}$ *m* without P3HT.

The optical properties of the silver bismuth iodide samples were determined by measuring the transmittance and reflectance. Figure 2 shows the absorbance (*A*), *A*=100−*R*(*λ*)−*T*(*λ*) (*R* is the reflectance and *T* is the transmittance) in the 300–1600 nm range.

**Figure 2:**
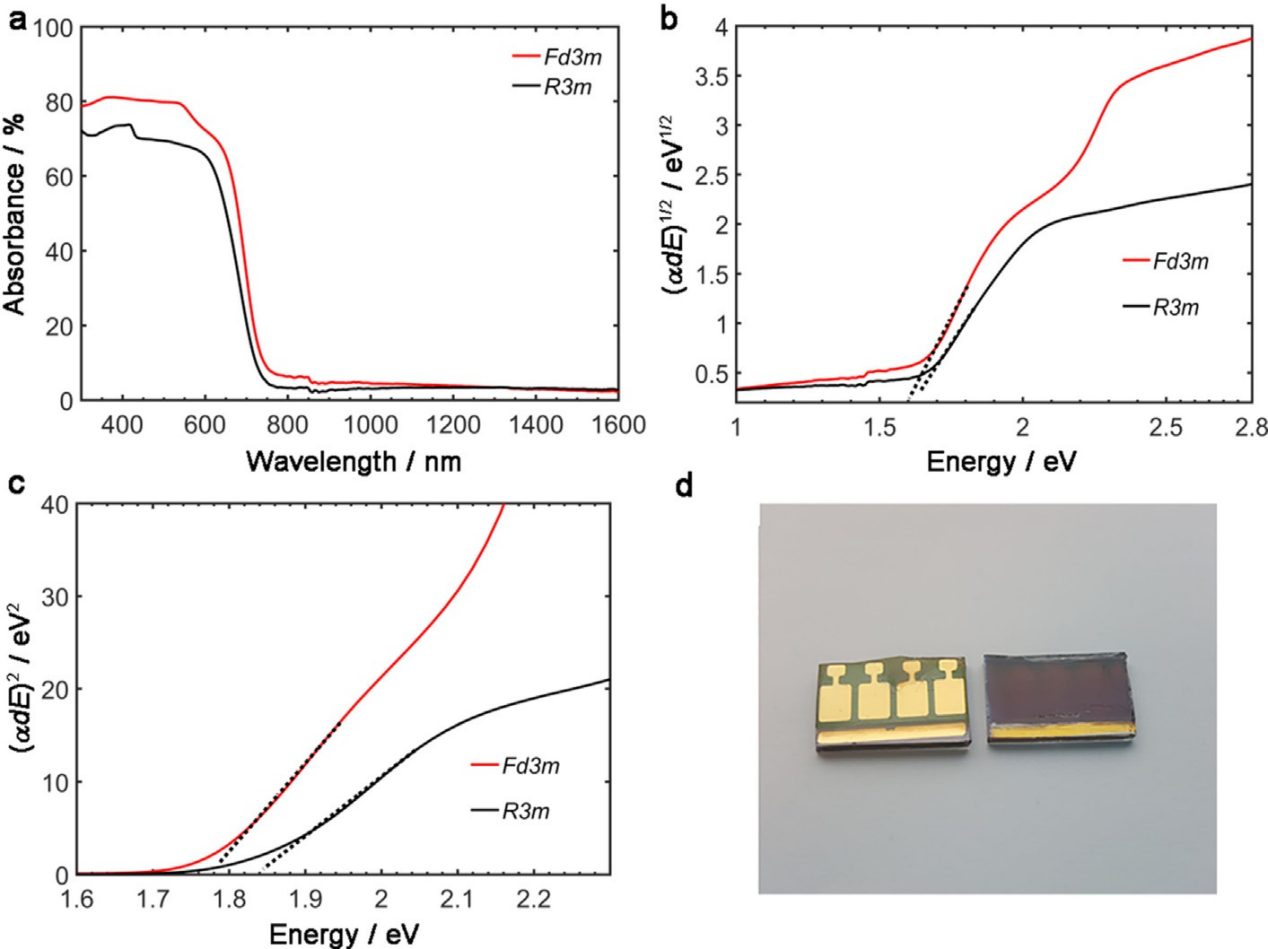
UV/Vis spectra from measured transmittance and reflectance of the samples with space group *Fd* $\bar{3}$ *m* and *R* $\bar{3}$ *m*, with molar ratios AgI/BiI_3_=1:2 and AgI/BiI_3_=2:1, respectively. a) Absorbance together with b) indirect and c) direct optical transitions estimated from the absorption coefficient *α*. d) Picture of devices based on silver bismuth iodide with space group *R* $\bar{3}$ *m* with molar ratio AgI/BiI_3_=2:1.

Both samples show similar character of spectra with absorption of 70–80 % above the band gap. The sample with space group *Fd* $\bar{3}$ *m* has slightly higher absorption throughout the spectrum. Tauc plots were implemented in Figure 2 b, c to experimentally determine the indirect and direct optical band gaps. In the expression (*α d E*)^*r*^, *α* corresponds to the absorption coefficient and *d* the thickness of the thin film (here constant) and *E* is the photon energy. In the expression, *r*=1/2 for an indirect allowed band gap, and *r*=2 for a direct allowed band gap. From this expression the Tauc plots in Figure 2 b and c are obtained; using a linear extraction, in Figure 2 b the indirect band gaps are determined and in Figure 2 c the direct band gaps are determined. The band gap (*E*_g_) of the indirect optical transitions is estimated to be1.60 eV for the *Fd* $\bar{3}$ *m* sample and 1.62 eV for the *R* $\bar{3}$ *m* sample. The direct optical band gap energies are determined to be 1.78 eV for the *Fd* $\bar{3}$ *m* sample and 1.85 eV for the *R* $\bar{3}$ *m* sample.

The surface morphology of the crystallized films annealed at 150° C was characterized using SEM. Figure 3 shows SEM images of the two samples: a) *Fd* $\bar{3}$ *m* with grain size 400–800 nm and b) *R* $\bar{3}$ *m* with grain size 500–900 nm. The grains have grown densely together even if pinholes could be observed especially in Figure 3 a. In Figure 3 b also small light particles are observed, which can be observed in some areas of this sample (see also the Supporting Information). To analyze the composition of the materials and the light particles, energy‐dispersive X‐ray spectroscopy (EDX) was used. When using EDX a higher acceleration voltage 20 kV was necessary to obtain the elemental composition, and we observed that the material and the light particles changed during the measurement, and finally the light particles disappeared after around 5 min of measurement. EDX measurements are therefore not completely reliable due to the change in the material during measurements, and the EDX results (Supporting Information) show that the silver and iodide content is less than expected. It is possible that reactions occur during which silver and iodide ions are lost from the surface. Small particles have lower melting point than the bulk material, which may increase the possibility for material changes during the measurement with the high‐intensity electron beam.^32^, ^33^ XRD patterns before and after the SEM and EDX measurements are shown in Figure S3, which shows that the sample structure is also changed. At lower magnification, the *Fd* $\bar{3}$ *m* sample in Figure 3 c shows two types of grains. The square‐shaped grains are displayed in Figure 3 a at higher magnification and the other type of grains shown in Figure 3 c are more flat grains, sticking up from the surface (also shown in Figure S4). This indicates two types of materials that coexist in the thin film. The sample with space group *R* $\bar{3}$ *m* in Figure 3 d has only one type of grain, however, the surface coverage is not 100 % and the TiO_2_ can be observed at a few places most probably leading to short circuit in the solar cell at these points.

**Figure 3:**
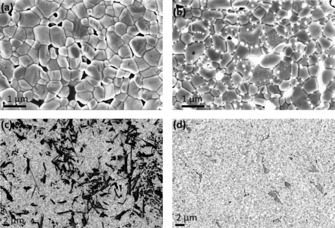
SEM images of the surface morphology of samples with space group a) *Fd* $\bar{3}$ *m* (AgI/BiI_3_=1:2), b) *R* $\bar{3}$ *m* (AgI/BiI_3_=2:1) at lower magnification, c) *Fd* $\bar{3}$ *m* sample with lower magnification, d) *R* $\bar{3}$ *m* sample with lower magnification.

Figure 4 shows the results from the photovoltaic characterization of solar cells based on Ag_2_BiI_5_ and AgBi_2_I_7_ (device structure in Figure 4 d). Figure 4 a shows the IPCE spectra (also called external quantum efficiency, EQE) of solar cells based on the materials with *R* $\bar{3}$ *m* and *Fd* $\bar{3}$ *m* space group structures. The IPCE onset for both devices is around 700 nm, which is in agreement with the UV/Vis spectra. The maximum IPCE value for the device with *R* $\bar{3}$ *m* space group structure is about 45 %, which is around 4 times higher than for the device with *Fd* $\bar{3}$ *m* space group structure, and the IPCE shows a good reproducibility for several devices (Figure S7). Comparing to previously reported devices based on bismuth‐halide light absorbers, the sample with space group *R* $\bar{3}$ *m* shows significantly higher IPCE. The reason for the higher photoconversion efficiency is not clear at the moment, but it may be related to more efficient electron transport to TiO_2_, or longer lifetime of the photogenerated charges in the sample with space group *R* $\bar{3}$ *m* under different light intensity (Figure 4 b), which results in higher photocurrent. Figure 4 c shows the current–voltage characteristics of the devices under illumination. Comparing the devices, a PCE of 2.1 % was obtained for the device based on the material with space group *R* $\bar{3}$ *m*, and a PCE of 0.4 % was obtained for the device based on the material with space group *Fd* $\bar{3}$ *m*. The higher photocurrent for the device based on the material with space group *R* $\bar{3}$ *m* is in agreement with the results from the IPCE spectra. The reproducibility of the solar cells is rather good, and for a series of devices based on the material with space group *R* $\bar{3}$ *m* an average short‐circuit current (*J*_sc_) of around 5.4 mA cm^−2^ is obtained, and the average PCE is around 1.5 % (see Figure S8). The stability of the champion device was also investigated. The sample was kept under N_2_ environment in dark and the performance of the device shows almost the same efficiency after 40 days, with a slight loss (0.2 %) of efficiency, which is mainly owed to photocurrent loss (Figure S7, left). These promising results exemplify the possibility to tune the device and material performance of new lead‐free metal halides by changing the stoichiometry of the components, yielding different crystal structure and different optoelectronic properties.

**Figure 4:**
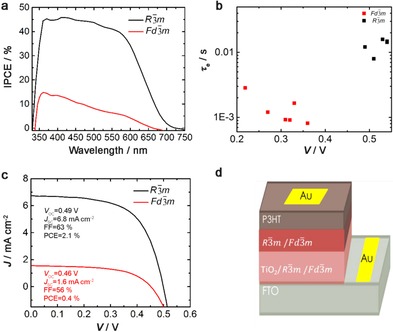
a) IPCE spectrum of the samples with space group *R* $\bar{3}$ *m* and *Fd* $\bar{3}$ *m*, b) electron lifetime, c) IV measurements, d) schematic of the device architecture.

In summary, we have characterized the lead‐free hexagonal metal‐halide with space group *R* $\bar{3}$ *m* as light absorber in solar cells, and compared with the previously reported AgBi_2_I_7_ sample with space group on *Fd* $\bar{3}$ *m*. Both the materials with space group *R* $\bar{3}$ *m* and *Fd* $\bar{3}$ *m* have an indirect band gap and a direct band gap with slightly higher energy. The *R* $\bar{3}$ *m*‐based devices showed a maximum incident photon‐to‐current efficiency (IPCE) of 45 % and the *Fd* $\bar{3}$ *m*‐based devices showed a maximum IPCE of around 11 %. The device with the material with *R* $\bar{3}$ *m* space group structure shows a very promising power conversion efficiency (PCE) above 2 %. The results show the potential for finding new lead‐free metal‐halides and the possibility to tune the properties of bismuth halides adding different amounts of silver ions and different structures. In a future perspective, the large variety of possible metal‐halide materials, varying the elements, stoichiometry, and structure in metal‐halides, makes it therefore likely to find several materials with advantageous photovoltaic properties.

## Experimental Section

### Materials

BiI_3_ (Sigma–Aldrich 99.99 %), AgI (Sigma–Aldrich 99 %), and P3HT (Sigma–Aldrich 99.995 %) were purchased and used as supplied. The solvent for precursors was butylamine (Sigma–Aldrich 99.5 %). The P3HT was dissolved in chlorobenzene (Sigma–Aldrich 99.8 %), the concentration of P3HT is 10 mg mL^−1^.

### Fabrication of solar cells

The forming of TiO_2_ blocking layer and mesoporous TiO_2_ layer are the same was done by an etching method previously reported by our group.^15^ The molar ratios of AgI and BiI_3_ used were 1:2 and 2:1, the concentration of all solutions was 17 wt %. Precursor solutions were spin‐coated on the TiO_2_ substrate at 4000 rpm for 30 s under flow of N_2_. After heating at 150 °C for 30 min in a dry box (relative humidity is around 20 %), the light‐absorbing films were formed. Then, P3HT was spin‐coated on the top of light‐absorbing films under 3000 rpm for 30 s. Last, 80 nm‐thick gold electrode was evaporated on P3HT layers by thermal evaporation under the vacuum of about 10^−5^ mbar.

### Characterization

**XRD**: The structure of the metal‐iodide materials were determined by grazing incidence X‐ray diffraction (GIXRD), using a Siemens D5000 *θ*‐2 *θ* goniometer with CuK_α_ (*λ*=1.54051 Å) radiation and 0.4° Soller‐slit collimator that had a resolution of 0.3° (2 *θ*) (Bruker AXS, Karlsruhe, Germany).

**PCE**: The photovoltaic performance of cells were recorded by using a Keithley 2400 source meter with a scan rate of 50 mV s^−1^ under AM 1.5 G (1000 W⋅m^−2^) illumination with a solar simulator (Model: 91160), which was calibrated with a standard Si solar cell (Fraunhofer ISE), and the power supplier was Newport Oriel(Model: 69911). The solar cells were masked during the measurement and the active area was defined as 0.125 cm^2^.

**IPCE**: The IPCE spectra were recorded using a Keithley multimeter (Model 2700) as a function of wavelength of the light from 350 to 900 nm. A monochromator (Spectral Products, CM 110) was used to obtain monochromatic light. The setup was calibrated with a standard Si solar cell (Fraunhofer ISE) prior to measurements. All solar cells were illuminated from the working electrode (glass substrate) side with an active area of 0.125 cm^2^ (circular shaped mask).

**Electron lifetime measurements**: Electron lifetime was determined by transient photovoltage decay at different light intensities. The source was a white LED (ST‐210WHF, Seventeam). The voltage was recorded with a 16‐bit resolution digital acquisition board (BNC‐2110, National Instruments) with a current amplifier (Stanford Research Systems, SR570).

**SEM measurements**: The cross section of the solar cells was measured with SEM using a LEO 1550 FEG instrument (LEO Electron Microscopy Ltd., Cambridge, UK) with in‐lens detector.

**UV/Vis**: The optical reflectance and transmittance of the samples were measured with a PerkinElmer Lambda 900 double‐beam UV/Vis/NIR spectrophotometer equipped with an integrating sphere and a Spectralon reflectance standard. The absorbance *A*(*λ*) was measured by using the following equation: *A*(*λ*)=100−*T*(*λ*)−*R*(*λ*), *T*(*λ*) is the transmittance and *R*(*λ*) is the reflectance.

SEM images, EDX measurements, XRD patterns, histograms of parameters of Ag_2_BiI_5_ solar cells; *J*_sc_, *V*_oc_, fill factor (FF), and PCE for 20 solar cells, *I*–*V* measurement, IPCE measurement, and the photo of precursor solution, are provided in the Supporting Information.

## Conflict of interest

*The authors declare no conflict of interest*.

## Supporting information

As a service to our authors and readers, this journal provides supporting information supplied by the authors. Such materials are peer reviewed and may be re‐organized for online delivery, but are not copy‐edited or typeset. Technical support issues arising from supporting information (other than missing files) should be addressed to the authors.

Supplementary
