# Supplementary material for: High Photon‐to‐Current Conversion in Solar Cells Based on Light‐Absorbing Silver Bismuth Iodide
Source: ChemSusChem. 2017 Jun 1;10(12):2592–6. doi: 10.1002/cssc.201700634 (PMC5499729; doi:10.1002/cssc.201700634)
Supplement: Supplementary file 1 — Supplementary [file CSSC-10-2592-s001.pdf]

CHEMISTRY & SUSTAINABILITY
CHEMSUSCHEM
ENERGY & MATERIALS

Supporting Information

High Photon-to-Current Conversion in Solar Cells Based on Light-Absorbing Silver Bismuth Iodide

Huimin Zhu, Mingao Pan, Malin B. Johansson, and Erik M. J. Johansson*[a]

cssc_201700634_sm_miscellaneous_information.pdf

Author Contributions

The manuscript was written by the contributions of H. Z., M. B. J. and E M. J. J. H. Z., M. B. J. and M. P. cooperated in all experimental parts. All authors have given approval to the final version of the manuscript.

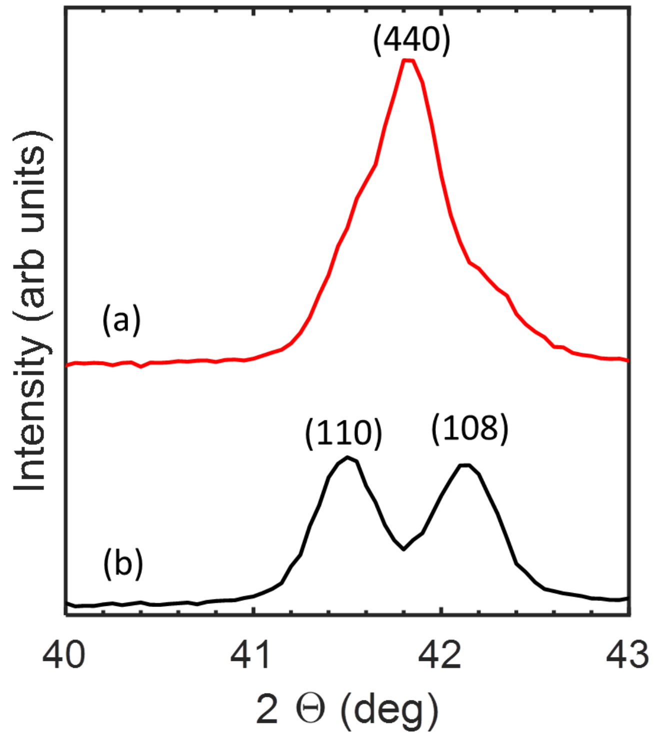

Figure S1 XRD pattern 40-43° 2θ (a) of a $AgI:BiI_3$ sample with space group *Fd*3*m,* with a diffraction plane (440), the reference card nr 00-034-1372 (b) of a $AgI:BiI_3$ sample with space group *R3m,* with two diffraction planes (110) and (108), the reference card nr 00-035-1025

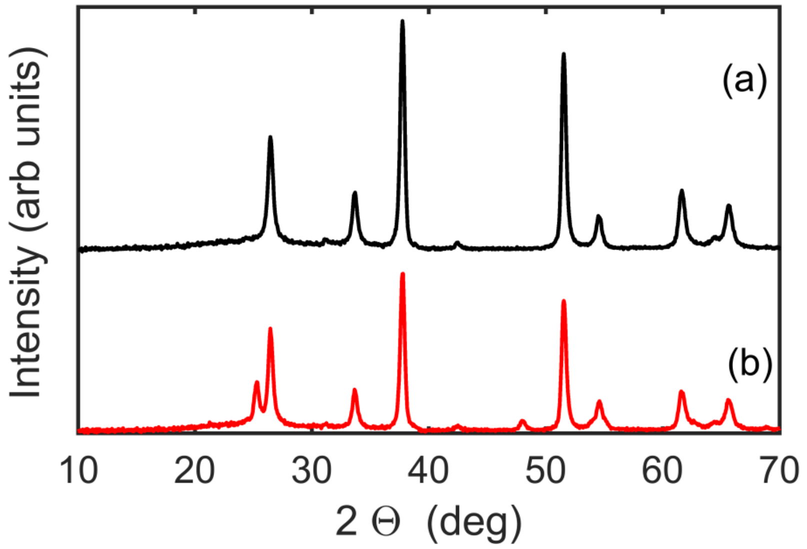

Figure S2 XRD pattern on (a) FTO glass and (b) FTO glass with TiO_2

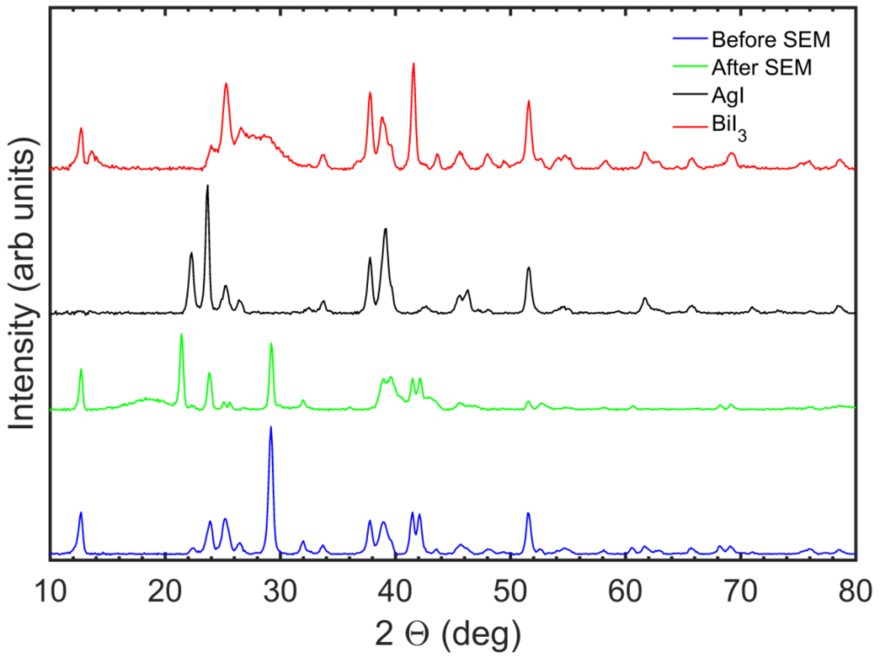

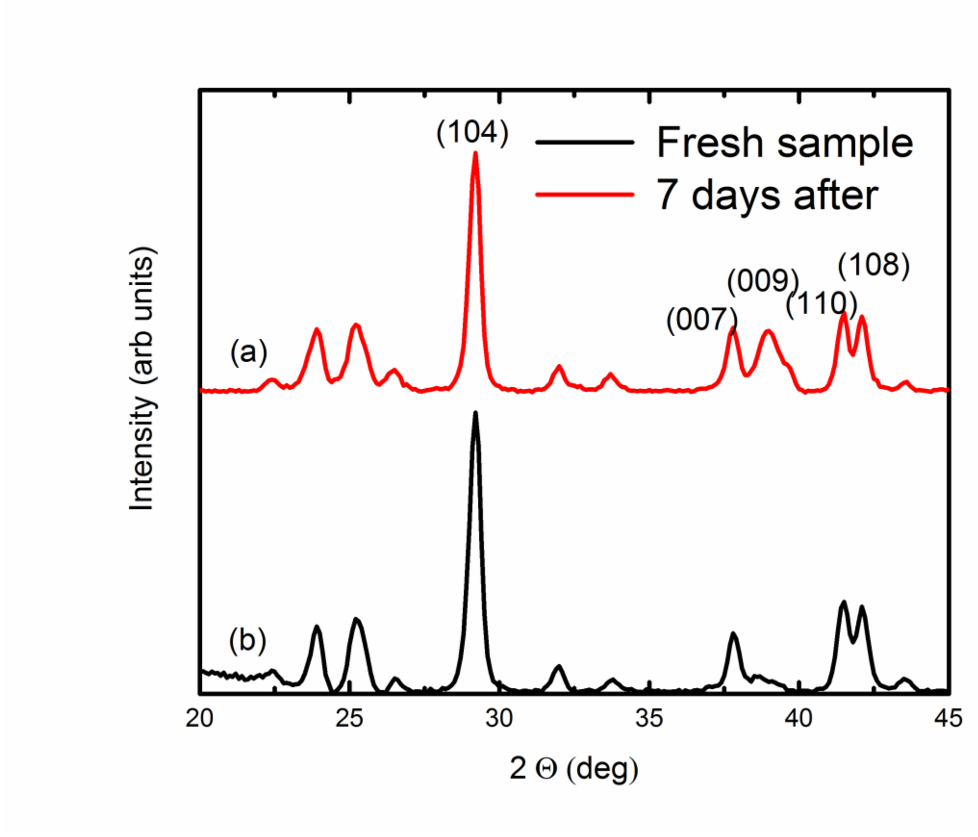

Figure S3 XRD pattern of a $AgI:BiI_3$ sample with space group R3m before and after SEM measurement (top) and the film stability characterization of the sample with space group R3m

for a fresh sample and a 7 days old sample stored under nitrogen (bottom). References for AgI PDF card No. 00-009-0374 and BiI3 PDF card No. 01-074-0457.

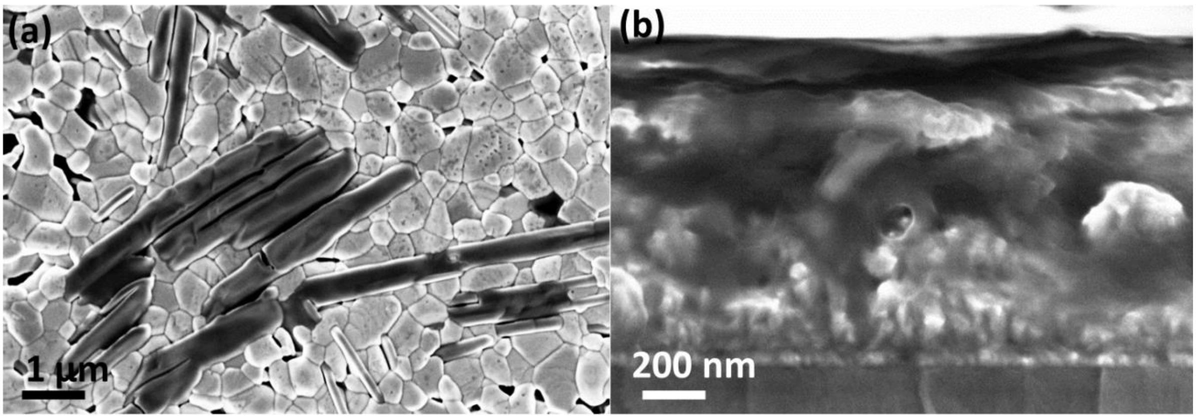

Figure S4 SEM image of (a) the (1:2) sample with two types of structure and (b) the cross section of a AgI:BiI_3 sample with space group $R3m$

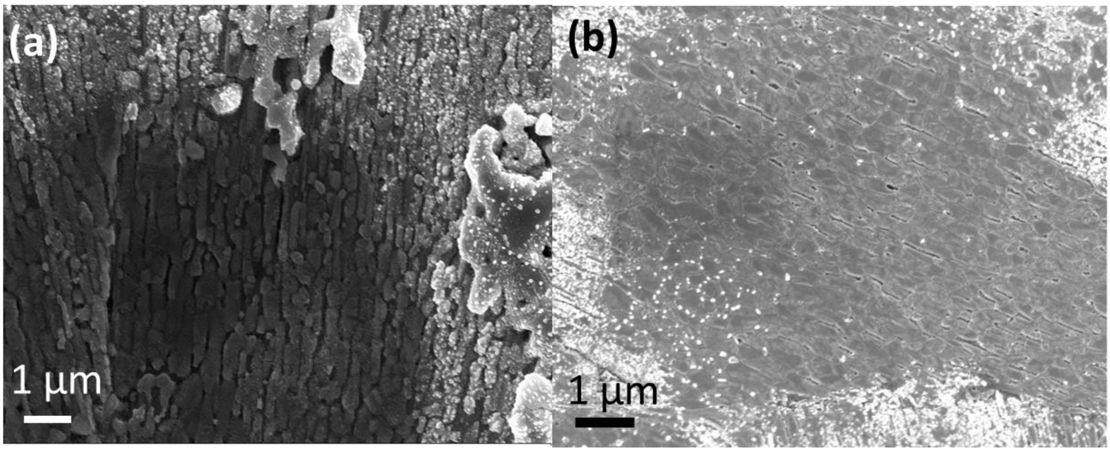

Figure S5 SEM image of the $AgI:BiI_3$ sample with space group *R*3*m*, a zoom out from a smaller area used in EDX analysis showing that the bright dots disappear after exposure to energetic electrons from high acceleration voltage EHT = 20 kV, during SEM analysis EHT ≈ 3.16 kV.

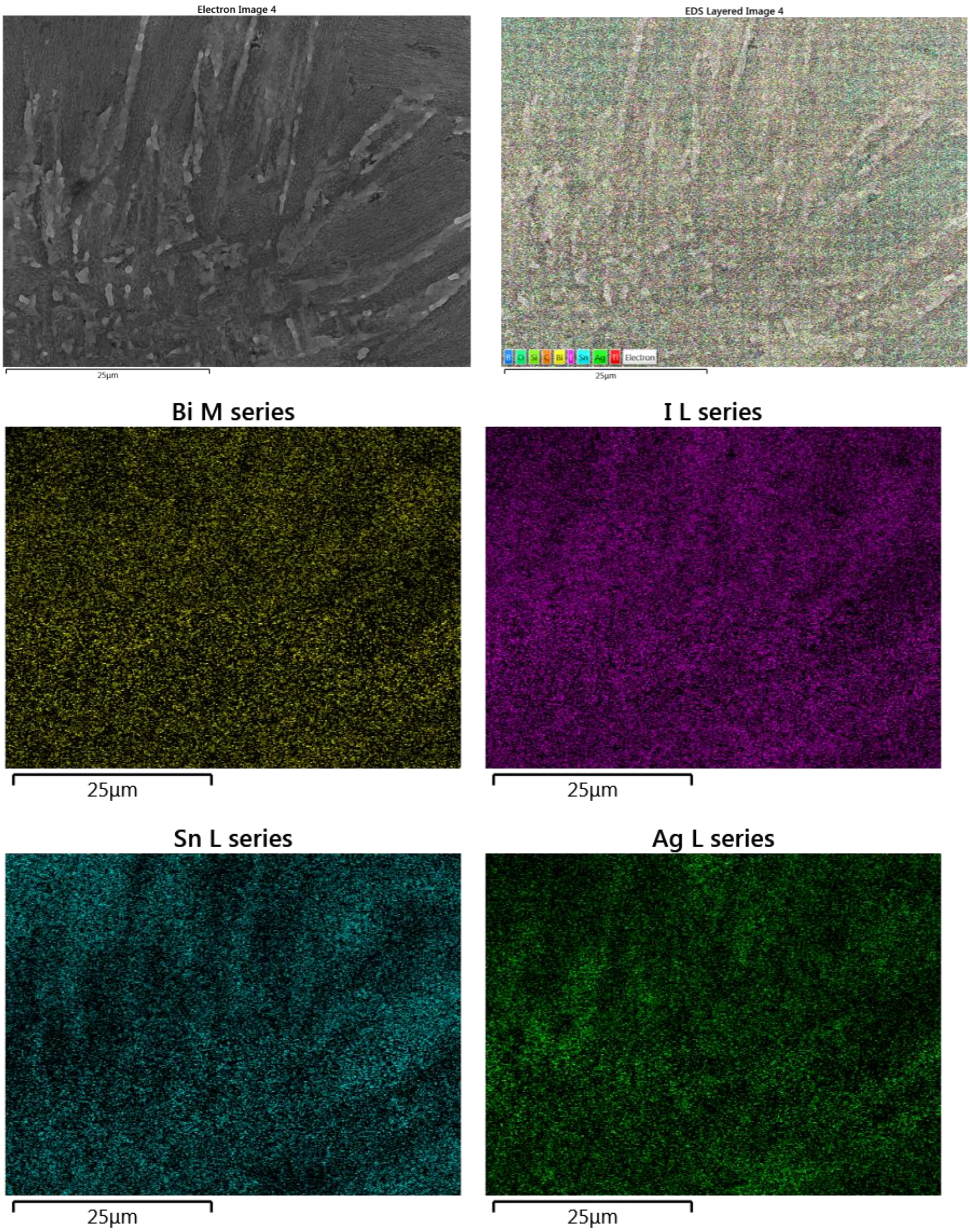
Electron Image 4
25µm
EDS Layered Image 4
25µm
Bi M series
25µm
I L series
25µm
Sn L series
25µm
Ag L series
25µm

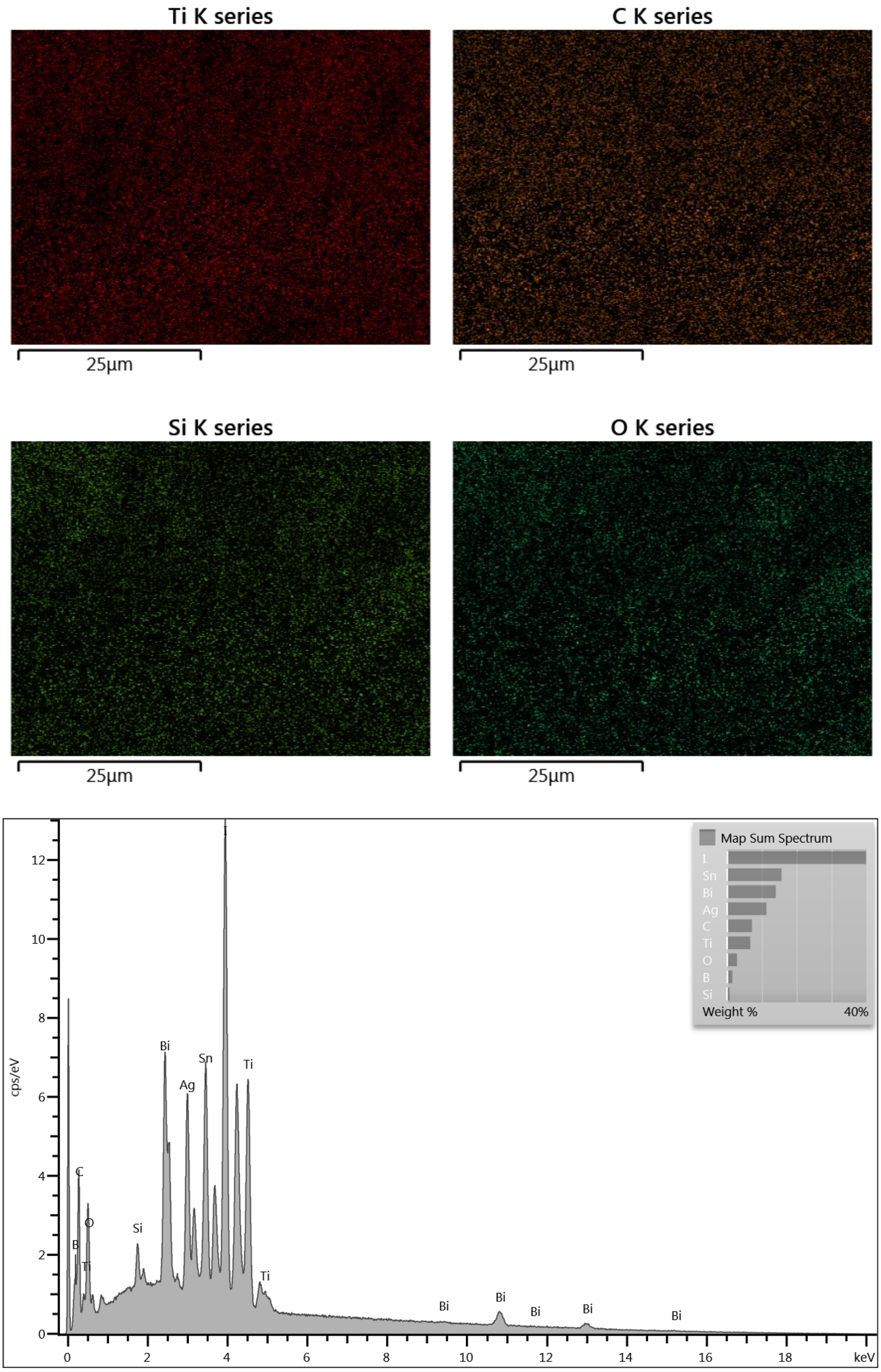
Ti K series
25µm
C K series
25µm
Si K series
25µm
O K series
25µm
Map Sum Spectrum
I
Sn
Bi
Ag
C
Ti
O
B
Si
Weight %
40%
cps/eV
0
2
4
6
8
10
12
0
2
4
6
8
10
12
14
16
18
keV
I
Bi
Sn
Ag
Ti
C
O
B
Ti
Si
Ti
Bi
Bi
Bi
Bi
Bi

Figure S6 EDX measurement of the AgI:BiI$_3$ sample with space group *R*3*m*, showing the elements occurring at the sample.

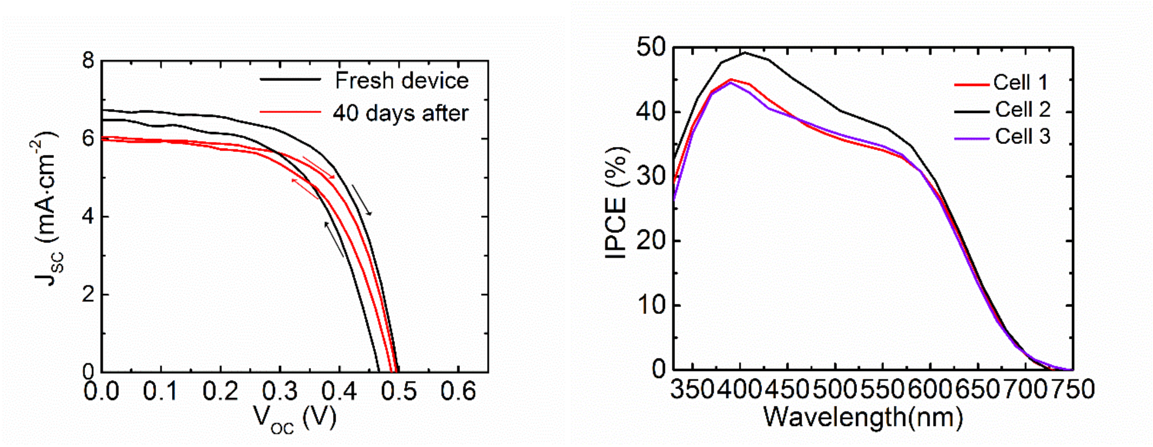

Figure S7 The champion device's forward and reverse scan of the 1st and 40th day (left) and three IPCE measurements of different solar cells based on the same material.

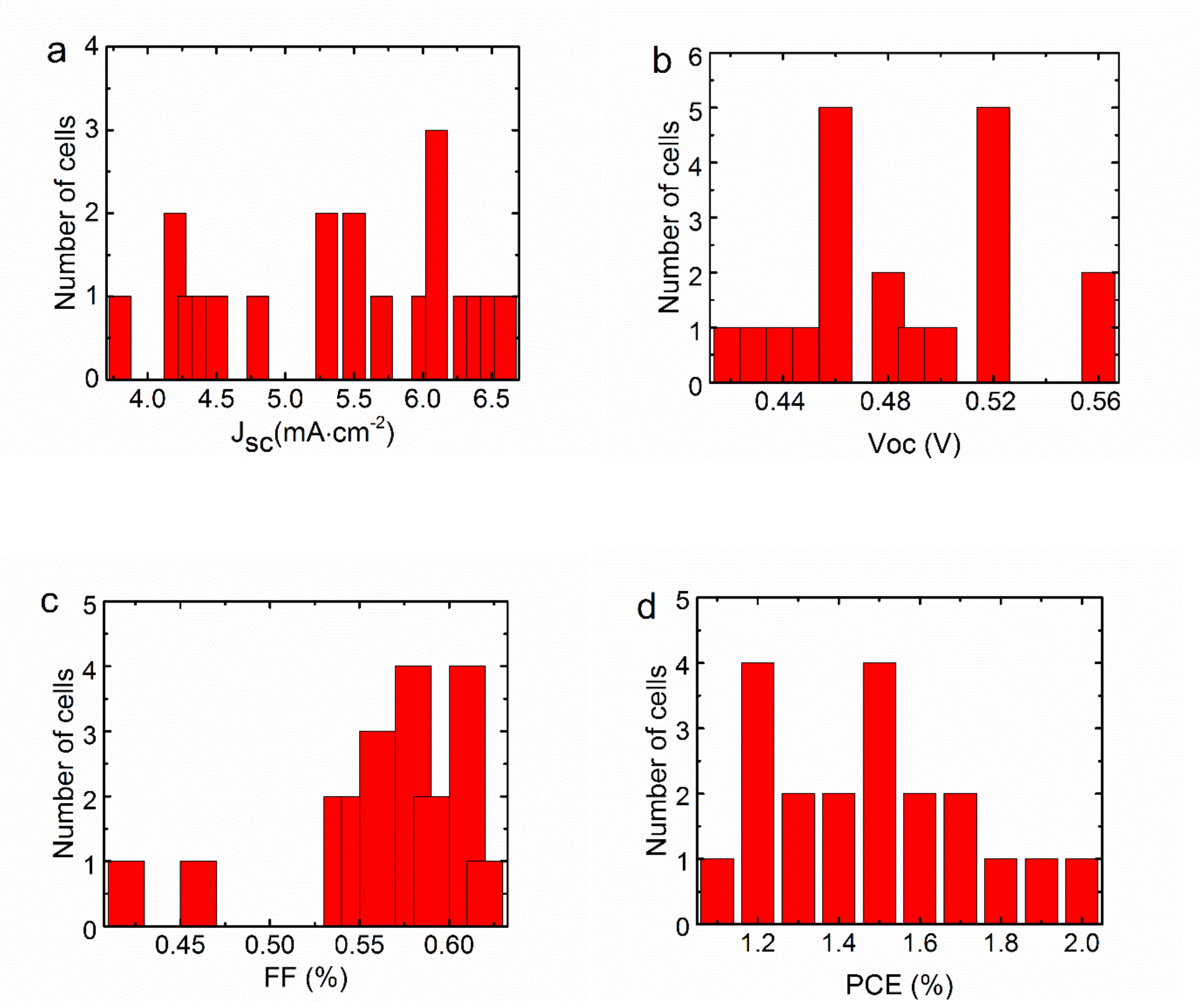

Figure S8 The histograms of parameters of AgI:BiI_3 with space group *R*3*m* based solar cells. (a) Jsc, (b) Voc, (c) FF, and (d) PCE for 20 solar cells, respectively.

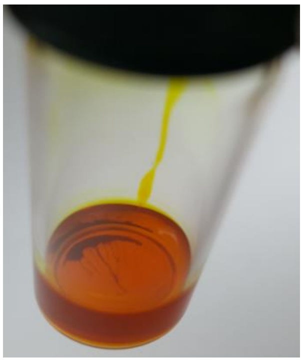

Figure S9 The AgI:BiI_3; 2:1 molar ratio solution.
